# Identifying patients with atrial fibrillation during sinus rhythm on ECG: Significance of the labeling in the artificial intelligence algorithm

**DOI:** 10.1016/j.ijcha.2022.100954

**Published:** 2022-01-11

**Authors:** Shinya Suzuki, Jun Motogi, Hiroshi Nakai, Wataru Matsuzawa, Tsuneo Takayanagi, Takuya Umemoto, Naomi Hirota, Akira Hyodo, Keiichi Satoh, Takayuki Otsuka, Takuto Arita, Naoharu Yagi, Takeshi Yamashita

**Affiliations:** a Department of Cardiovascular Medicine, The Cardiovascular Institute, Tokyo, Japan; b Nihon Kohden Corporation, Tokyo, Japan; c Information System Division, The Cardiovascular Institute, Tokyo, Japan

**Keywords:** Atrial fibrillation, Artificial intelligence, Electrocardiography

## Abstract

**Background:**

This study aimed to increase the knowledge on how to enhance the performance of artificial intelligence (AI)-enabled electrocardiography (ECG) to detect atrial fibrillation (AF) on sinus rhythm ECG (SR-ECG).

**Methods:**

It is a retrospective analysis of a single-center, prospective cohort study (Shinken Database). We developed AI-enabled ECG using SR-ECG to predict AF with a convolutional neural network (CNN). Among new patients in our hospital (n = 19,170), 276 AF label (having ECG on AF [AF-ECG] in the ECG database) and 1896 SR label with following three conditions were identified in the derivation dataset: (1) without structural heart disease, (2) in AF label, SR-ECG was taken within 31 days from AF-ECG, and (3) in SR label, follow-up ≥ 1,095 days. Three patterns of AF label were analyzed by timing of SR-ECG to AF-ECG (before/after/before-or-after, CNN algorithm 1 to 3). The outcome measurement was area under the curve (AUC), sensitivity, specificity, accuracy, and F1 score. As an extra-testing dataset, the performance of AI-enabled ECG was tested in patients with structural heart disease.

**Results:**

The AUC of AI-enabled ECG with CNN algorithm 1, 2, and 3 in the derivation dataset was 0.83, 0.88, and 0.86, respectively; when tested in patients with structural heart disease, 0.75, 0.81, and 0.78, respectively.

**Conclusion:**

We confirmed high performance of AI-enabled ECG to detect AF on SR-ECG in patients without structural heart disease. The performance enhanced especially when SR-ECG after index AF-ECG was included in the algorithm, which was consistent in patients with structural heart disease.

## Highlights

• High performance of AI algorithm to detect AF using SR-ECG was confirmed in patients without structural heart disease.
• The performance of AI-enabled ECG to detect AF was high especially when the algorithm included SR-ECG taken after the index AF-ECG.
• A similar tendency was observed when the performance was tested in patients with structural heart diseases.

## 1 Introduction

Atrial fibrillation (AF) is among the most common cardiac rhythm disorders and is associated with increased morbidity (e.g., ischemic stroke) and mortality. One major challenge is promptly diagnosing AF after onset because of its silent nature in many patients. As tools for screening AF, many devices have been proposed over the gold standard tool of 12-lead electrocardiography (ECG), such as patient-initiated devices (oscillometric blood pressure cuff, intermittent ECG rhythm strip, or photoplethysmogram on smartphone), semi-continuous (smart watch ECG) or continuous wearable devices (long-term Holter, wearable belts, or 1–2 week continuous ECG patches), and implanted devices [1], [2]. In patients with cryptogenic stroke, in which the origin of the thrombus is unknown, continuous and repeated monitoring with implanted or wearable devices have demonstrated a substantial burden of undiagnosed AF [3], [4].

In practical viewpoint, simple methods to discriminate patients at a high risk of AF would help to identify candidates for such long-term monitoring device. For example, precise analysis of waveforms of resting 12-lead ECG using artificial intelligence (AI) has identified patients with AF on sinus rhythm ECG (SR-ECG) [5]. This method is unique because, although the existence of AF on 12-lead ECG is a gold standard for diagnosing AF, this method by AI gains insight into the existence of AF on ECG in which AF is absence. Attia et al. [5] reported a landmark study of AI-enabled ECG to predict AF on ECG with SR from the Mayo Clinic, and Raghunath et al. [6] reported AI-enabled ECG from the Geisinger Health System with a larger ECG database. These studies had a strong impact for their high predictive ability, with an area under the curve (AUC) of around 0.900.

After the publishment of these studies, additional important tasks in the methodologies have raised, one being labeling problems. For example, among ECG recordings with the AF label, ECG recordings in SR had different predictive abilities according to the length of time to incident AF [5], [7]. Moreover, there remain tasks in the methodologies of AI-enabled ECG which have not been fully investigated. First, the performance of AI-enabled ECG may differ depending on the existence of structural heart disease because typical ECG findings may mask refined AF-related features on SR-ECG. Second, whether SR-ECG for developing AI-enabled ECG should be taken before or after the index AF-ECG is unknown. Even when AF has never been detected, undetected AF may already exist. Therefore, AI-enabled ECG may need the information on SR-ECG after AF occur. Third, the performance of AI-enabled ECG in the SR-label may be different according to the length of the observation period in the database because, simply, it is assumed that the shorter the time-period of apparently keeping sinus rhythm, the lower the certainty of the absence of undetected AF.

In the present study, we developed AI-enabled ECG within SR-ECG to predict AF using a single-center ECG database to enhance the performance of AI-enabled ECG in special reference to these unresolved issues.

## 2 Methods

### 2.1 Ethics and informed consent

This study was performed in accordance with the Declaration of Helsinki (revised in 2013) and Ethical Guidelines for Medical and Health Research Involving Human Subjects (Public Notice of the Ministry of Education, Culture, Sports, Science and Technology, and the Ministry of Health, Labour and Welfare, Japan, issued in 2017). Written informed consent was obtained from all participants. The study protocol was reviewed by the Institutional Review Board of the Cardiovascular Institute.

### 2.2 Identifying the study groups

#### 2.2.1 Total study population

**Fig. 1:**
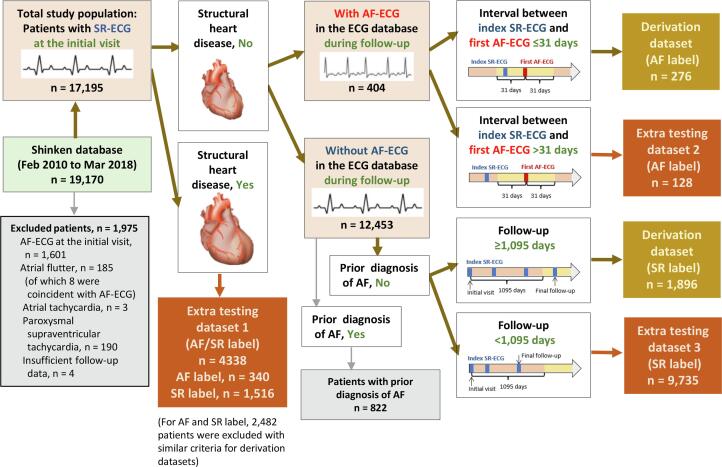
Flowchart of patient selection. SR, sinus rhythm; ECG, electrocardiography; AF, atrial fibrillation.

The Shinken database includes all patients who newly visited the Cardiovascular Institute, Tokyo, Japan, excluding foreign travelers and patients with active cancer. This single-hospital database was established in June 2004. Details of this database have been described elsewhere [8]. In the present study, 19,170 patients registered between February 2010 and March 2018 were extracted from the Shinken database because a computerized electrocardiogram database has been available since February 2010. We excluded 1975 patients for one or more of the following reasons: AF on ECG at the initial visit (n = 1601), atrial flutter (n = 185; of which 8 were coincident with AF), atrial tachycardia (n = 3), paroxysmal supraventricular tachycardia (n = 190), and insufficient follow-up data (n = 4). The remaining 17,195 patients with SR-ECG were the target of the present study (Fig. 1).

#### 2.2.2 Study population for development and evaluation of AI-enabled ECG (derivation dataset)

Out of 17,195 patients in the total study population, 2172 patients were selected as the derivation dataset which comprised 276 patients with AF label and 1896 patients with SR label (Fig. 1). In the present study, SR-ECGs were used for the analysis, where they were assigned to “AF label” when at least one ECG showing AF (AF-ECG) was found in the same patient in the ECG database during the follow-up, while they were assigned to “SR label” when no AF-ECG was found during the follow-up.

As shown in the flowchart (Fig. 1), patients with AF label in the derivation dataset (1) did not have structural heart disease, (2) had at least one AF-ECG in the ECG database during follow-up, and (3) had at least one SR-ECG within 31 days before or after the first AF-ECG. Meanwhile, patients with SR label in the derivation dataset (1) did not have structural heart disease, (2) had no AF-ECG in the ECG database during follow-up, (3) did not have prior diagnosis of AF before the first visit to our hospital, and (4) having an observation period ≥ 1095 days.

#### 2.2.3 Study population for testing the performance of AI-enabled ECG (extra testing dataset)

Among the patients who were excluded in the process of selecting patients for derivation dataset, followings were defined as extra testing datasets and used for testing the performance of AI-enabled ECG (Fig. 1): (1) patients with structural heart disease (extra testing dataset 1; n = 4338), out of which 340 AF label and 1516 SR label were identified with similar exclusion criteria as for the derivation dataset, (2) patients who did not have structural heart disease, had at least one AF-ECG in the ECG database during follow-up, and did not have SR-ECG within 31 days before or after the first AF-ECG (extra testing dataset 2; n = 128), and (3) patients who did not have structural heart disease, had no AF-ECG in the ECG database during follow-up, did not have prior diagnosis of AF before the first visit to our hospital, and had an observation period < 1095 days (extra testing dataset 3; n = 9735).

The patients in the extra testing dataset 2 were further divided according to the timing of the index SR-ECG from the first AF-ECG (Fig. 3): ≤−366 days (n = 85), −365 to −181 days (n = 62), −180 to −91 days (n = 49), −90 to –32 days (n = 43), 32 to 90 days (n = 55), 91 to 180 days (n = 74), 181 to 365 days (n = 87), and ≥ 366 days (n = 93). The patients in the extra testing dataset 3 were further divided according to the length of observation period (Fig. 3): ≤180 days (n = 8,013), 181 to 365 days (n = 370), 366 to 730 days (n = 821), and 731 to 1094 days (n = 531).

### 2.3 Data sampling

Twelve-lead ECG was recorded for 10 s in the supine position using an ECG machine (GE CardioSoft V6.71 and MAC 5500 HD; GE Healthcare, Chicago, IL, USA) at a sampling rate of 500 Hz, and raw data of digital records were stored using the MUSE data management system. Out of each 10-second ECG recording, 5-second ECG samples were extracted. The reason for sampling with a 5-second duration was because we employed oversampling to balance the number of samples between AF and SR labels [9], [10]. The details of oversampling (the scientific background and the detail process of oversampling) are explained in the Supplementary document.

#### 2.3.1 Derivation dataset

In patients with the SR label, the index SR-ECG for the analysis was the one obtained at the initial visit. In the SR label, each 10-second ECG was divided at the half which yielded two samples of 5-second ECGs.

**Fig. 2:**
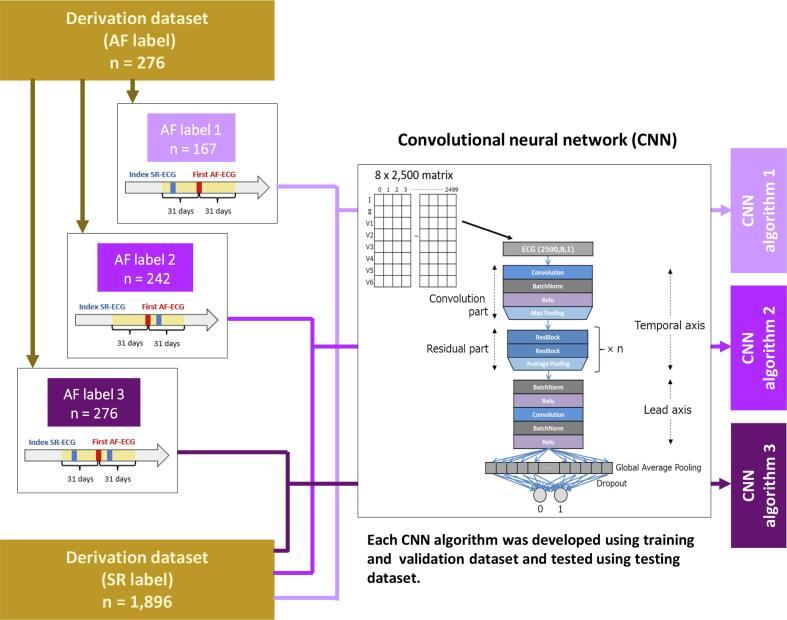
Convolutional neural network (CNN) analysis. For each AF label, the index SR-ECG was chosen within 31 days before the first AF-ECG (AF label 1), within 31 days after the first AF-ECG (AF label 2), or within 31 days before or after the first AF-ECG (AF label 3). Using the AF label 1, 2, and 3, CNN algorithm 1, 2, and 3, respectively, were developed combined with fixed SR label. AF, atrial fibrillation; SR, sinus rhythm; ECG, electrocardiography.

In patients with the AF label, the index SR-ECG was the one nearest (within 31 days) the first AF-ECG in the ECG database. Here, the index SR-ECG with the AF label was chosen according to three patterns: the pre- (AF label 1, n = 167), post- (AF label 2, n = 242), or pre- or post- (AF label 3, n = 276) 31-day period of the first AF-ECG (Fig. 2). As three patterns of AF label were defined, three patterns of derivation dataset (SR label/AF label 1, SR label/AF label 2, and SR label/AF label 3) were consequently yielded (Fig. 2). In the AF label, 5-second ECGs were obtained to balance the number of samples between AF and SR labels by the data augmentation with sliding window (Supplementary Fig. 1).

**Fig. 3:**
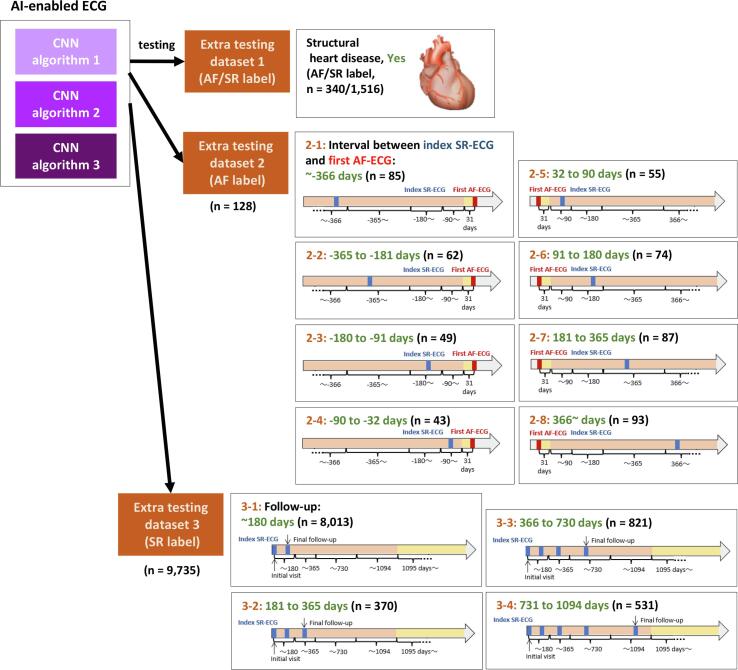
Detail categorization of the Extra testing datasets. The patients in the extra testing dataset 2 were further divided according to the timing of the index SR-ECG from the first AF-ECG. The patients in the extra testing dataset 3 were further divided according to the length of observation period. The number of patients in each detail category was for whom ECG was taken in each time category. CNN, convolutional neural network; AF, atrial fibrillation; SR, sinus rhythm; ECG, electrocardiography.

In each derivation dataset, ECG samples were divided into the training, validation, and testing datasets at a ratio of 7:1:2 (details of the number of samplings are displayed in Supplementary Fig. 2A).

#### 2.3.2 Extra testing dataset

In patients in the Extra testing datasets, each 10-second ECG was divided at the half which yielded two samples of 5-second ECGs per one 10-second ECG (details of the number of samplings are displayed in Supplementary Fig. 2B).

### 2.4 AI-enabled ECG

#### 2.4.1 Convolutional neural network (CNN) modeling

We constructed a CNN using the Keras Framework with a Tensorflow (Google; Mountain View, CA, USA) backend and Python. Of the eight physical leads and four augmented leads with a 10-second duration on 12-lead electrocardiography (ECG) recordings, we selected the eight independent leads (leads I, II, and V1–6) with a 5-second duration. Accordingly, the original 12 × 5000 matrix (i.e., 12 leads with a 10-second duration sampled at 500 Hz) was reduced to an 8 × 2500 matrix.

The CNN model had layers for a temporal axis and a lead axis [5]. The layers for the temporal axis were composed of two parts: the convolution part and the residual part. The convolution part included a convolution layer, a batch-normalization layer, a layer for non-linear Rectified Linear Unit (ReLU) activation, and a maximum pooling layer [11]. The residual part included a combination of two residual blocks based on Residual Network (ResNet) [12] and average pooling, which was repeated N times, and the value of N was tuned to obtain the best performance (the method is outlined below). The layers for the lead axis were composed of a paired batch-normalization layer and a layer for non-linear ReLU activation, followed by a convolution layer. Thereafter, a second paired batch-normalization layer and a layer for non-linear ReLU activation were included. Finally, the data were fed to a dropout layer with global average pooling and to the final output layer activated by the softmax function, which generated the probability of AF. The architecture of the model is shown in Fig. 1B.

The model was trained on a computer with 192-GB RAM and single Quadro P-2200 (NVIDIA) graphics processing units that were used to train the model using Keras.

A receiver operating characteristic (ROC) curve was created to validate and test the data to assess the AUC of AI-enabled ECG to determine whether AF was present. Using the ROC curve in the validation dataset, we tuned the number of repetitions for the combination of the two residual blocks and average pooling written above (N). Moreover, we determined the probability threshold of AF using the ROC curve. These parameters were used for the final evaluation using the testing dataset.

#### 2.4.2 Outcome measurement

The primary outcome of the study was the ability of AI-enabled ECG to identify patients with AF using SR-ECG, which was assessed by the AUC, sensitivity, specificity, accuracy, and F1 score of the model. In the derivation dataset and the extra testing dataset 1, the AUC, sensitivity, specificity, accuracy, and F1 score of the model were assessed. In the extra testing dataset 2 and 3, only the accuracy was tested because the datasets were consisted of single label (AF and SR label, respectively).

We used two-sided 95% confidence intervals (CIs) to summarize sample variability in the estimates. We used exact CIs (Clopper–Pearson) to be conservative for accuracy, sensitivity, and specificity. The CIs for the AUC were estimated using Sun’s and Su’s optimization of the Delong method using the pROC package [13], whereas the CIs for F1 were obtained using the bootstrap method with 2000 replications. Analyses of exact CIs were performed using Python version 3.7.6 (Python Software Foundation, DE, USA), and other analyses were performed using R version 4.0.3 (The R Foundation, Vienna, Austria).

## 3 Results

### 3.1 Development of AI-enabled ECG in the derivation datasets

Among all included patients in the derivation dataset (n = 2172; AF label, n = 276; SR label, n = 1896), the mean age was 60.1 ± 13.6 years at the initial visit, and 1170 patients (53.9%) were male.

**Fig. 4:**
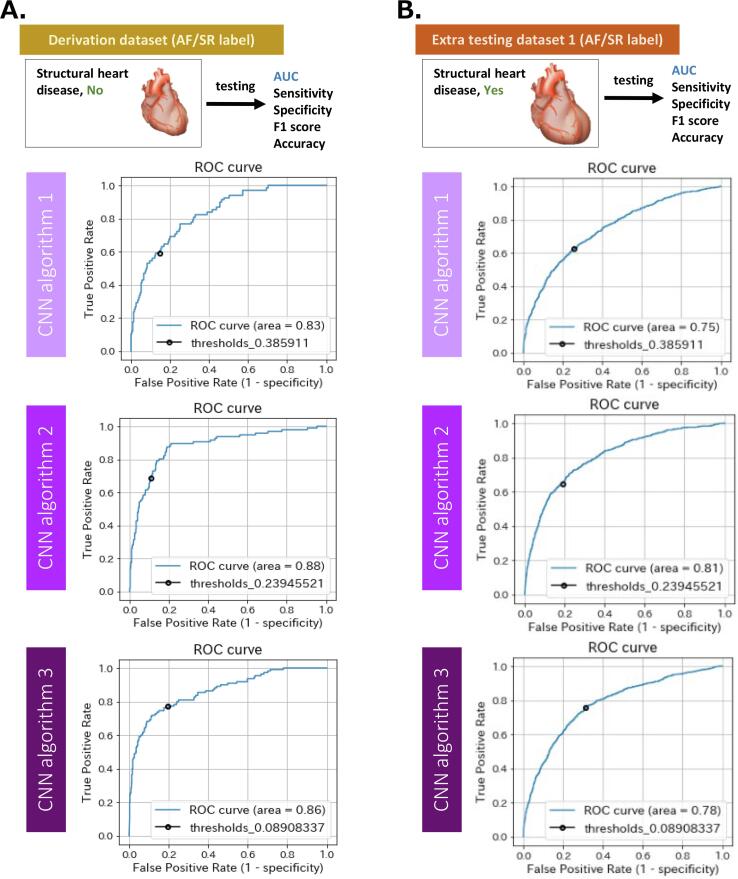
ROC curves for prediction of AF by AI-enabled ECG using SR-ECG. A. Derivation dataset. B. Extra testing dataset 1. The detail measurement of the performance is displayed in Table 1. ROC, receiver operating characteristic; AF, atrial fibrillation; AI, artificial intelligence; ECG, electrocardiography; SR, sinus rhythm.

The performance of AI-enabled ECG for CNN algorithm 1, 2, and 3 are shown in Table 1A and Fig. 4A. The AUC of AI-enabled ECG was 0.83 (0.78–0.88) for CNN algorithm 1, 0.88 (0.84–0.92) for CNN algorithm 2, and 0.86 (0.82–0.90) for CNN algorithm 3. The accuracy was 0.83 (0.81–0.86) for CNN algorithm 1, 0.87 (0.84–0.89) for CNN algorithm 2, and 0.79 (0.76–0.82) for CNN algorithm 3. The performance was mostly similar between CNN algorithm 2 and 3, which was a little bit higher than that of CNN algorithm 1.

**Table 1:** Performance of AI-enabled ECG predicting atrial fibrillation by SR-ECG in derivation dataset and extra testing dataset 1.

A. Derivation dataset					
	AUC (95% CI)	Sensitivity (95% CI)	Specificity (95% CI)	F1 score (95% CI)	Accuracy (95% CI)
CNN algorithm 1	0.83 (0.78 – 0.88)	0.59 (0.46 – 0.71)	0.86 (0.83 – 0.88)	0.37 (0.29 – 0.45)	0.83 (0.81 – 0.86)
CNN algorithm 2	0.88 (0.84 – 0.92)	0.69 (0.58 – 0.78)	0.89 (0.87 – 0.91)	0.54 (0.47 – 0.62)	0.87 (0.84 – 0.89)
CNN algorithm 3	0.86 (0.82 – 0.90)	0.77 (0.68 – 0.85)	0.79 (0.76 – 0.82)	0.48 (0.42 – 0.55)	0.79 (0.76 – 0.82)

B. Extra testing dataset 1					
	AUC (95% CI)	Sensitivity (95% CI)	Specificity (95% CI)	F1 score (95% CI)	Accuracy (95% CI)
CNN algorithm 1	0.75 (0.72 – 0.77)	0.62 (0.58 – 0.66)	0.74 (0.72 – 0.76)	0.42 (0.39 – 0.45)	0.72 (0.71 – 0.74)
CNN algorithm 2	0.81 (0.79 – 0.83)	0.65 (0.61 – 0.68)	0.81 (0.80 – 0.83)	0.51 (0.48 – 0.54)	0.78 (0.77 – 0.80)
CNN algorithm 3	0.78 (0.76 – 0.80)	0.76 (0.72 – 0.79)	0.69 (0.67 – 0.70)	0.48 (0.45 – 0.51)	0.70 (0.68 – 0.71)

AI, artificial intelligence; ECG, electrocardiography; AUC, area under the curve; CNN, convolutional neural network.

The ROC curves are presented in Fig. 4.

### 3.2 Testing the performance of AI-enabled ECG in the extra testing datasets

The results of testing the performance of AI-enabled ECG for CNN algorithm 1, 2, and 3 in the extra testing dataset 1 are shown in Table 1B and Fig. 4B. The AUC of AI-enabled ECG was 0.75 (0.72–0.77) for CNN algorithm 1, 0.81 (0.79–0.83) for CNN algorithm 2, and 0.78 (0.76–0.80) for CNN algorithm 3. The accuracy was 0.72 (0.71–0.74) for CNN algorithm 1, 0.78 (0.77–0.80) for CNN algorithm 2, and 0.70 (0.68–0.71) for CNN algorithm 3. The performance of AI-enabled ECG in the extra testing dataset 1 was a little bit lower than that in the derivation dataset, whereas their patterns in the three CNN algorithms were similar to those in the derivation dataset.

**Fig. 5:**
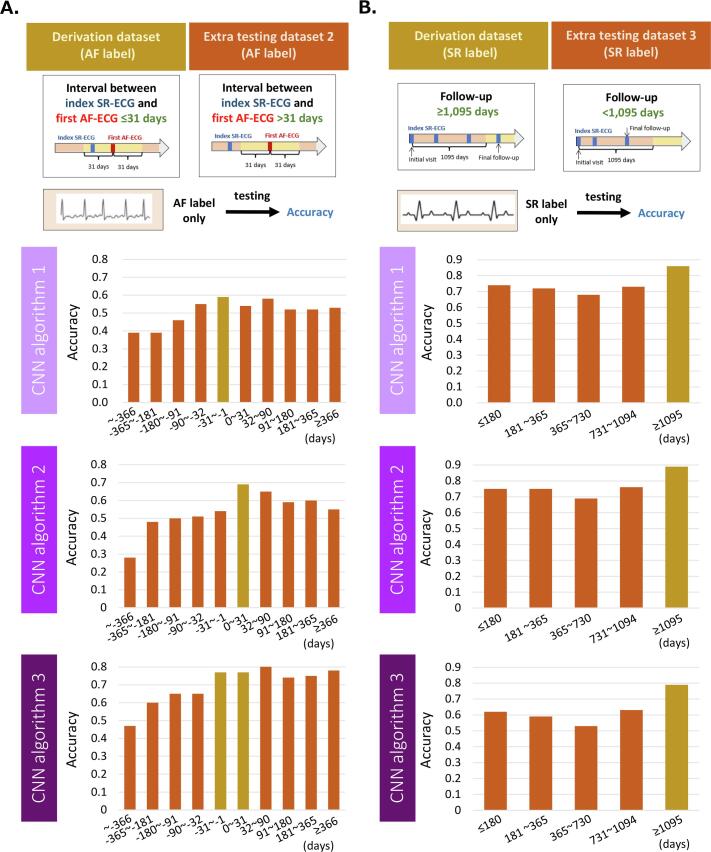
Accuracy of AI-enabled ECG for predicting (A) AF in extra testing dataset 2 and (B) SR in extra testing dataset 3. Detail data are shown in Table 2.

The results of testing the performance of AI-enabled ECG for CNN algorithm 1, 2, and 3 in the extra testing dataset 2 are shown in Table 2A and Fig. 5A. Commonly among the three CNN algorithms, when SR-ECG was taken before AF-ECG, the accuracy of AI-enabled ECG increased according to the timing of SR-ECG became close to AF-ECG. Meanwhile, when SR-ECG was taken after AF-ECG, the accuracy of AI-enabled ECG was mostly similar irrespective of the length of time between SR-ECG and AF-ECG. The accuracy of AI-enabled ECG was generally higher in CNN algorithm 3 compared with that in CNN algorithm 1 and 2.

**Table 2:** Performance of AI-enabled ECG predicting atrial fibrillation by SR-ECG in extra testing dataset 2 and 3.

A. Extra testing dataset 2			
Length of time from the index AF-ECG	CNN algorithm 1	CNN algorithm 2	CNN algorithm 3
	Accuracy (95% CI)	Accuracy (95% CI)	Accuracy (95% CI)
<−366 days	0.39 (0.32 – 0.47)	0.28 (0.22 – 0.36)	0.47 (0.39 – 0.55)
−365 to −181 days	0.39 (0.30 – 0.48)	0.48 (0.39 – 0.58)	0.60 (0.50 – 0.68)
−180 to −91 days	0.46 (0.36 – 0.56)	0.50 (0.40 – 0.60)	0.65 (0.55 – 0.75)
−90 to –32 days	0.55 (0.44 – 0.65)	0.51 (0.40 – 0.62)	0.65 (0.54 – 0.75)
−31 to −1 days	**0.59 (0.46 – 0.71)**	0.54 (0.44 – 0.63)	**0.77 (0.68 – 0.85)**
0 to 31 days	0.54 (0.48 – 0.60)	**0.69 (0.58 – 0.78)**	
32 to 90 days	0.58 (0.48 – 0.68)	0.65 (0.55 – 0.73)	0.83 (0.74 – 0.89)
91 to 180 days	0.52 (0.44 – 0.60)	0.59 (0.51 – 0.67)	0.74 (0.66 – 0.81)
181 to 365 days	0.52 (0.45 – 0.60)	0.60 (0.52 – 0.67)	0.75 (0.68 – 0.81)
≥366 days	0.53 (0.45 – 0.60)	0.55 (0.47 – 0.62)	0.78 (0.72 – 0.84)

B. Extra testing dataset 3			
Length of observation period	CNN algorithm 1	CNN algorithm 2	CNN algorithm 3
	Accuracy (95% CI)	Accuracy (95% CI)	Accuracy (95% CI)
≤180 days	0.74 (0.73 – 0.74)	0.75 (0.75 – 0.76)	0.62 (0.61 – 0.63)
181 to 365 days	0.72 (0.68 – 0.75)	0.75 (0.71 – 0.78)	0.59 (0.56 – 0.63)
366 to 730 days	0.68 (0.66 – 0.70)	0.69 (0.66 – 0.71)	0.53 (0.50 – 0.55)
731 to 1094 days	0.73 (0.71 – 0.76)	0.76 (0.73 – 0.78)	0.63 (0.60 – 0.66)
≥1095 days	**0.86 (0.83 – 0.88)**	**0.89 (0.87 – 0.91)**	**0.79 (0.76 – 0.82)**

Bold numbers with shadow indicate the data of the derivation dataset. Given that the accuracy for the single label (AF label in extra testing dataset 2 and SR label in extra testing dataset 3) was equal to sensitivity (for AF label) or specificity (for SR label), the bold numbers in the Table 2A and 2B were the sensitivity and the specificity, respectively, in the derivation dataset for each CNN algorithm.

These data are visualized in Fig. 5.

CNN, convolutional neural network; AF, atrial fibrillation; SR, sinus rhythm.

The results of testing the performance of AI-enabled ECG for CNN algorithm 1, 2, and 3 in the extra testing dataset 3 are shown in Table 2B and Fig. 5B. Commonly among the three CNN algorithms, the accuracy of AI-enabled ECG was mostly similar irrespective of the length of the observation period. The accuracy of AI-enabled ECG was generally higher in CNN algorithm 1 and 2 compared with that in CNN algorithm 3.

## 4 Discussion

### 4.1 Major findings

In the present study, we developed AI-enabled ECG to predict AF using 12-lead SR-ECG in patients without structural heart disease by three patterns according to the timing of the index SR-ECG, and thereafter confirmed the performance in patients with structural heart disease. The AUC of AI-enabled ECG was higher when the algorithm included SR-ECG taken after the AF-ECG (0.88 and 0.86 for CNN algorithm 2 and 3 compared to 0.83 for CNN algorithm 1). Similar tendency was observed when the AI-enabled ECG was tested in patients with structural heart disease (0.81 and 0.78 for CNN algorithm 2 and 3 compared to 0.75 for CNN algorithm 1).

### 4.2 Comparison with previous studies

AI-enabled ECG to predict AF using 12-lead SR-ECG has already been reported by multiple study groups [5], [6], [7]. These groups reported a high predictive ability for AF using the AUC, which was 0.90 in the study by Attia et al. and 0.87 in the study by Raghunath et al. It was quite surprising that SR-ECG can predict AF with such a high predictive capability. In the present study, we obtained a similar AUC of 0.88 and 0.86 when SR-ECG in AF label was taken after and before/after, respectively, the index AF-ECG.

Such studies were based on the hypothesis that the AF signature due to structural changes in the atria can be identified by 12-lead ECG during SR [5], [14] because structural changes in the atria predispose to atrial arrhythmia [15]. Moreover, in our previous study using hundreds of ECG parameters analyzed with the random forest algorithm, the importance of ECG parameters in predicting AF was similar in the P wave, QRS complex, and ST-T segment, which suggested that structural changes in the ventricle, presumably due to aging or atherosclerosis, seem to be similarly important [16].

### 4.3 Characteristics and clinical implications of the AI-enabled ECG in the present study

Attia et al. [5] enrolled 180,922 patients with 649,931 SR-ECGs in their landmark paper, and Raghunath et al. [6] enrolled 430,909 patients with 16,234,87 ECG recordings in their prospective study. However, in the present study, we used only 276 and 1896 patients for AF-label and SR-label, respectively, with, at most, 2,994 and 3,792 ECG samples, which attained AUC over 0.8 and near to 0.9. Of course, it is not surprising that our AI-enabled ECG showed a high performance with a small number of sampling because we simplified our model by 1) excluding patients with structural heart disease, 2) restricting SR-ECG with the AF label to patients within 31 days from the first AF-ECG, 3) restricting SR-ECG with the SR label to patients with a follow-up period of ≥ 1,095 days, and 4) taking a balance of the number of samples using an over sampling method. Of note, through our model, we believe we can learn some points how to increase the performance of AI-enabled ECG to predict AF on SR-ECG.

First, the timing of SR-ECG to AF-ECG in AF-label would be important. In the present study, we developed three patterns of AI-enabled ECG deriving from three patterns of AF-label, where the index SR-ECG was taken before, after, or before-or-after the first AF-ECG (CNN algorithm 1, 2, and 3, respectively). When we compared their performance, the AUC was higher when the algorithm included SR-ECG taken after the AF-ECG (0.88 and 0.86 for CNN algorithm 2 and 3) than when it did not (0.83 for CNN algorithm 1). Based on the result, we can learn two points: (1) AI-enabled ECG can detect the structural changes in atrium before AF incidence because the AUC was over 0.8 even when the model included only the information before the first AF-ECG (CNN algorithm 1), and (2) AI-enabled ECG enhanced the performance remarkably when the information after the first AF-ECG was included (the AUC increased near to 0.9 in CNN algorithm 2 and 3). These results are supported by previous clinical reports. In a study that investigated the changes in atrium between before and after direct cardioversion for AF, left atrial dimension did not decrease at 1 week after the cardioversion (before and after cardioversion, 44.8 mm and 44.3 mm) [17]. Meanwhile, another similar study showed that left atrial volume index decreased at 1 month after cardioversion (41.12 mL/m^2^ and 37.56 mL/m^2^) [18]. Therefore, significant structural changes occur in atrium when AF happens and they may still remain within 1 week even after the cardiac rhythm restores to the sinus rhythm [17] but remarkably improved at 1 month [18]. On the other hand, in a sub-analysis of the Multi-Ethnic Study of Atherosclerosis study, an increment of left atrial volume index or a decrease of left atrial physiological function over time predict the incidence of AF [19]. It would be an attractive concept that AI-enabled ECG can predict the future incidence of AF. But this concept is not so realistic. For example, although Attia et al. demonstrated a high AUC (0.900) with SR-ECG within 31 days of AF-ECG [5], the AUC decreased (0.71–0.73) for prediction after approximately 2 years and further decreased to ∼ 0.60 for prediction at ≥ 4 years [7]. On the other hand, in a practical viewpoint, AI-enabled ECG should have a more important role to detect the already-existent AF (sometimes, asymptomatic AF) with a high sensitivity. And, when the aim of AI-enabled ECG is to increase the sensitivity to detect the already-existent AF, our results suggest that the information of the SR-ECG after the first AF-ECG should be included in the development of AI-enabled ECG. In addition, when we tested the accuracy of the AI-enabled ECG in diagnosing AF on SR-ECG in a different timing from the first AF-ECG (Fig. 5), the accuracy was constantly high in CNN algorithm 3 (SR-ECG was taken both before and after AF-ECG). Therefore, to maximize the sensitivity to detect the already-existent AF among the three patterns of AF-label, the pattern of AF label in the CNN algorithm 3 seems to be the best.

Second, the tendency in the performance of three patterns of CNN algorithm in patients without structural heart disease was similar when they were applied to those with structural heart disease. Because patients with structural heart disease have various patterns of typical ECG features, especially in ST-T segment, we assumed that the existence of structural heart disease should increase the branches of patterns that AI-enabled ECG should learn and would affect its performance. Therefore, in the present study, by excluding patients with structural heart disease from our model, we intended to limit the variations in the derivation dataset. When we applied our model to patients with structural heart disease, the AUC was 0.75, 0.81, and 0.78 for CNN algorithm 1, 2, and 3, which were lower than the AUCs in patients without structural heart disease, but the tendency in the performance of three patterns of CNN algorithm was similar. Although the AUCs of AI-enabled ECG developed in patients without structural heart disease were lower in those with structural heart disease, they remained around 0.8, suggesting the AI-enabled ECG works to some extent beyond the absence or existence of structural heart disease. This may be because, even in patients with structural heart disease, typical ECG characteristics present only in limited patients with relatively severe conditions. These results possibly suggest that including patients with structural heart disease may not be a matter for developing AI-enabled ECG to detect AF on SR-ECG. Therefore, our model could be extrapolated to those with structural heart disease to some extent, but its utility requires further investigation.

The AI-enabled ECG to detect AF on SR-ECG may be a candidate to be incorporated in the technology tools supporting the guidelines-recommended integrated management of AF [1] by the following reasons. First, our findings suggest that AI-enabled ECG to predict AF on sinus-rhythm ECG can provide an aid for screening paroxysmal AF, especially in those who are strongly suspected of having AF (i.e., history of embolic stroke of undetermined sources and accumulation of risk factors for AF). Patients who have a high AF probability under the AI-enabled ECG would be candidates for screening AF more vigorously. Second, even in patients who are already diagnosed as paroxysmal AF, AI-enabled ECG to predict AF on sinus-rhythm ECG can provide information whether AF occurs recently (i.e., within 1 month). This would serve as simple information for managing AF patients under rhythm control therapy.

### 4.4 Limitations

There are several limitations of the present study that should be highlighted. First, although we limited SR-ECG recordings with the SR label to patients followed up for ≥ 1,095 days, there remained a possibility that undetected AF existed in patients with the SR label. Second, our study excluded patients with structural heart disease and can thus only be applied to similar populations. Third, our model of AI-enabled ECG should be verified against external datasets to confirm the generalizability.

### 4.5 Conclusions

We confirmed high performance of AI-enabled ECG to detect AF on SR-ECG in patients without structural heart disease. The performance enhanced especially when SR-ECG after index AF-ECG was included in the algorithm, which was consistent in patients with structural heart disease.

## Ethics and informed consent

This study was performed in accordance with the ethical norms based on the Declaration of Helsinki (revised in 2013) and Ethical Guidelines for Medical and Health Research Involving Human Subjects (Public Notice of the Ministry of Education, Culture, Sports, Science and Technology and the 10.13039/501100000008Ministry of Health, Labour and Welfare, Japan, issued in 2017). Written informed consent was obtained from all participants. The study protocol was reviewed by the Institutional Review Board of the Cardiovascular Institute.

## Consent for publication

Not applicable.

## Availability of data and materials

Data cannot be shared publicly because of lack of such a description in the study protocol and informed consent. Data are available from the Ethics Review Committee at the Cardiovascular Institute for researchers who meet the criteria for access to confidential data (contact via the corresponding author).

## Funding

This study was partially supported by the Practical Research Project for Life-Style related Diseases including Cardiovascular Diseases and Diabetes Mellitus from the Japan Agency for Medical Research and Development, AMED (JP17ek0210082).

## CRediT authorship contribution statement

**Shinya Suzuki:** Conceptualization, Validation, Writing – original draft. **Jun Motogi:** Conceptualization, Validation. **Hiroshi Nakai:** Conceptualization, Data curation, Validation. **Wataru Matsuzawa:** Data curation, Formal analysis. **Tsuneo Takayanagi:** Data curation, Formal analysis. **Takuya Umemoto:** Data curation, Formal analysis, Writing – original draft, Writing – review & editing. **Naomi Hirota:** Validation, Writing – review & editing. **Akira Hyodo:** Formal analysis, Validation. **Keiichi Satoh:** Formal analysis, Validation. **Takayuki Otsuka:** Writing – review & editing. **Takuto Arita:** Writing – review & editing. **Naoharu Yagi:** Writing – review & editing. **Takeshi Yamashita:** Funding acquisition, Writing – review & editing.

## Declaration of Competing Interest

The authors declare that they have no known competing financial interests or personal relationships that could have appeared to influence the work reported in this paper: [Dr. Suzuki received research funding from Mitsubishi Tanabe Pharm and Daiichi Sankyo. Dr. Yamashita received research funds and/or lecture fees from Daiichi Sankyo, Bayer Yakuhin, Bristol-Myers Squibb, Pfizer, Nippon Boehringer Ingelheim, Eisai, Mitsubishi Tanabe Pharm, Ono Pharmaceutical, and Toa Eiyo.].

## References

[1] Hindricks G., Potpara T., Dagres N. (2021). 2020 ESC Guidelines for the diagnosis and management of atrial fibrillation developed in collaboration with the European Association for Cardio-Thoracic Surgery (EACTS): The Task Force for the diagnosis and management of atrial fibrillation of the European Society of Cardiology (ESC) Developed with the special contribution of the European Heart Rhythm Association (EHRA) of the ESC. Eur. Heart J..

[2] January C.T., Wann L.S., Calkins H., Chen L.Y., Cigarroa J.E., Cleveland J.C., Ellinor P.T., Ezekowitz M.D., Field M.E., Furie K.L., Heidenreich P.A., Murray K.T., Shea J.B., Tracy C.M., Yancy C.W. (2019). 2019 AHA/ACC/HRS Focused Update of the 2014 AHA/ACC/HRS Guideline for the Management of Patients With Atrial Fibrillation: A Report of the American College of Cardiology/American Heart Association Task Force on Clinical Practice Guidelines and the Heart Rhythm Society in Collaboration With the Society of Thoracic Surgeons. Circulation.

[3] Sanna T., Diener H.-C., Passman R.S., Di Lazzaro V., Bernstein R.A., Morillo C.A., Rymer M.M., Thijs V., Rogers T., Beckers F., Lindborg K., Brachmann J. (2014). Cryptogenic stroke and underlying atrial fibrillation. N. Engl. J. Med..

[4] Gladstone D.J., Spring M., Dorian P., Panzov V., Thorpe K.E., Hall J., Vaid H., O'Donnell M., Laupacis A., Côté R., Sharma M., Blakely J.A., Shuaib A., Hachinski V., Coutts S.B., Sahlas D.J., Teal P., Yip S., Spence J.D., Buck B., Verreault S., Casaubon L.K., Penn A., Selchen D., Jin A., Howse D., Mehdiratta M., Boyle K., Aviv R., Kapral M.K., Mamdani M. (2014). Atrial fibrillation in patients with cryptogenic stroke. N. Engl. J. Med..

[5] Attia Z.I., Noseworthy P.A., Lopez-Jimenez F., Asirvatham S.J., Deshmukh A.J., Gersh B.J., Carter R.E., Yao X., Rabinstein A.A., Erickson B.J., Kapa S., Friedman P.A. (2019). An artificial intelligence-enabled ECG algorithm for the identification of patients with atrial fibrillation during sinus rhythm: a retrospective analysis of outcome prediction. Lancet.

[6] Raghunath S., Pfeifer J.M., Ulloa-Cerna A.E., Nemani A., Carbonati T., Jing L., vanMaanen D.P., Hartzel D.N., Ruhl J.A., Lagerman B.F., Rocha D.B., Stoudt N.J., Schneider G., Johnson K.W., Zimmerman N., Leader J.B., Kirchner H.L., Griessenauer C.J., Hafez A., Good C.W., Fornwalt B.K., Haggerty C.M. (2021). Deep Neural Networks Can Predict New-Onset Atrial Fibrillation From the 12-Lead ECG and Help Identify Those at Risk of Atrial Fibrillation-Related Stroke. Circulation.

[7] Christopoulos G., Graff-Radford J., Lopez C.L., Yao X., Attia Z.I., Rabinstein A.A., Petersen R.C., Knopman D.S., Mielke M.M., Kremers W., Vemuri P., Siontis K.C., Friedman P.A., Noseworthy P.A. (2020). Artificial Intelligence-Electrocardiography to Predict Incident Atrial Fibrillation: A Population-Based Study. Circ. Arrhythm Electrophysiol..

[8] Suzuki S., Yamashita T., Otsuka T., Sagara K., Uejima T., Oikawa Y., Yajima J., Koike A., Nagashima K., Kirigaya H., Ogasawara K., Sawada H., Aizawa T. (2011). Recent mortality of Japanese patients with atrial fibrillation in an urban city of Tokyo. J. Cardiol..

[9] Buda M., Maki A., Mazurowski M.A. (2018). A systematic study of the class imbalance problem in convolutional neural networks. Neural Netw..

[10] A. Le Guennec, S. Malinowski, R. Tavenard, Data Augmentation for Time Series Classification using Convolutional Neural Networks, in: ECML/PKDD Workshop on Advanced Analytics and Learning on Temporal Data. Riva Del Garda, Italy2016.

[11] J. Nagi, F. Ducatelle, A. Di Caro et al., Max-Pooling Convolutional Neural Networks for Vision-based Hand Gesture Recognition, in: IEEE International Conference on Signal and Image Processing Applicationss; Kuala Lumpur; Nov 16–18, 2011:342–47.

[12] K. He, X. Zhang, S. Ren, J. Sun, Deep Residual Learning for Image Recognition. arXiv pre-print server2015.

[13] Robin X., Turck N., Hainard A. (2011). pROC: an open-source package for R and S+ to analyze and compare ROC curves. BMC Bioinf..

[14] Hendriks J.M.L., Fabritz L. (2019). AI can now identify atrial fibrillation through sinus rhythm. Lancet.

[15] Kottkamp H. (2013). Human atrial fibrillation substrate: towards a specific fibrotic atrial cardiomyopathy. Eur. Heart J..

[16] Hirota N., Suzuki S., Arita T., Yagi N., Otsuka T., Kishi M., Semba H., Kano H., Matsuno S., Kato Y., Uejima T., Oikawa Y., Matsuhama M., Inoue T., Yajima J., Yamashita T. (2021). Prediction of current and new development of atrial fibrillation on electrocardiogram with sinus rhythm in patients without structural heart disease. Int. J. Cardiol..

[17] Yujing W., Congxin H., Shaning Y., Lijun J., Xiaojun H.u., Gang W.u., Qiang X. (2010). Digitalis does not improve left atrial mechanical dysfunction after successful electrical cardioversion of chronic atrial fibrillation. Cell Biochem. Biophys..

[18] Dell'Era G., Rondano E., Franchi E., Marino P.N. (2010). Atrial asynchrony and function before and after electrical cardioversion for persistent atrial fibrillation. Eur. J. Echocardiogr..

[19] Lim D.J., Ambale-Ventakesh B., Ostovaneh M.R., Zghaib T., Ashikaga H., Wu C., Watson K.E., Hughes T., Shea S., Heckbert S.R., Bluemke D.A., Post W.S., Lima J.A.C. (2019). Change in left atrial function predicts incident atrial fibrillation: the Multi-Ethnic Study of Atherosclerosis. Eur. Heart J. Cardiovasc. Imaging.

