# Supplementary material for: Identifying patients with atrial fibrillation during sinus rhythm on ECG: Significance of the labeling in the artificial intelligence algorithm
Source: Int J Cardiol Heart Vasc. 2022 Jan 11;38:100954. doi: 10.1016/j.ijcha.2022.100954 (PMC8760502; doi:10.1016/j.ijcha.2022.100954)
Supplement: Supplementary data 1 [file mmc1.pdf]

Supplementary File.

Contents

1. Oversampling 2

1.1. Reason why oversampling was employed in the present study 2

1.2. How oversampling was done.. 4

1. Oversampling

1.1. Reason why oversampling was employed in the present study

The reason for using oversampling in the present study was to balance the numbers of data between the two labels (SR and AF labels). The class imbalance problem in convolutional neural networks has been detected,[1] in which the imbalance of two labels affects the performance of the convolutional neural networks.

Oversampling is one of the methods to resolve the class imbalance problem. For example, in studies using time-series data,[2-4] oversampling was employed as a method of data augmentation, in which multiple data were obtained sliding in direction of the time axis. Similarly, in the present study, we obtained multiple data from a single 10-second ECG with Y-second sliding in direction of the time axis.

We obtained 5-second data out of each 10-second ECG. We also tried to cover the 10-second data by oversampling data. Accordingly, we obtained two 5-second samples out of 10-second ECG in SR label, in which two samples had no duplicated area and the total number of samples became twice as the number of 10-second ECGs in SR label. On the other hand, we obtained increased number of 5-second samples out of

10-second ECGs in AF label, in which the data had duplicated area with each other with Y-second slide so that the total area covers each 10-second ECG, and the number of samples was mostly equal to that of the samples taken in SR label. Thus, the former was the manner of oversampling for the label with large number (SR label, Pattern A) and the latter was the manner of oversampling for the label with small number (AF label, Pattern B) (Supplementary Figure 1).

For testing dataset (both main analysis and extra testing), the oversampling was done by Pattern B irrespective of AF or SR labels, because the class imbalance problem does not exist for testing dataset.

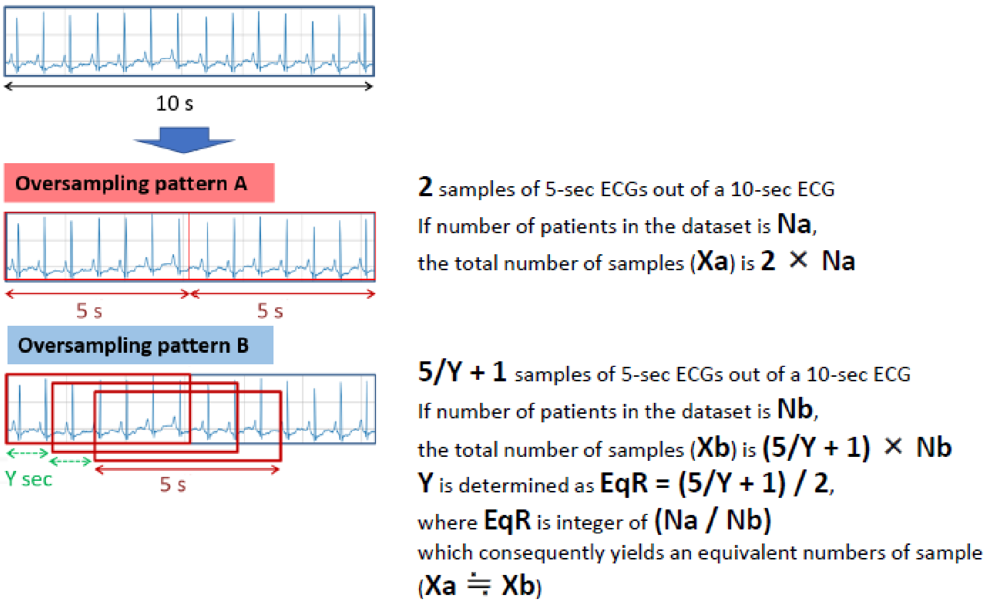

Supplementary Figure 1. Patterns of oversampling

1.2. How oversampling was done

In the manner of oversampling for the label with large number (typically, SR label in the derivation dataset, Pattern A), the 10-second ECG recordings were divided into two 5-second ECG samples. If number of patients in the dataset is Na, the total number of samples (Xa) is 2 multiplied by Na (Supplementary Figure 1).

In the manner of oversampling for the label with small number (AF label in the derivation dataset, Pattern B), multiple 5-second ECG samples were obtained by Y-second sliding in direction of the time axis. If number of patients in the dataset is Nb, Y is determined as $EqR = (5/Y + 1) / 2$, where EqR (equivalent rate) is an integer of (Na / Nb) which consequently yields equivalent numbers of sample (which means, Xa and Xb are nearly identical) (Supplementary Figure 1).

In the derivation dataset, ECG samples were divided into the training, validation, and testing datasets at a ratio of 7:1:2. The EqR for AF label 1, 2, and 3 in the derivation dataset was calculated as 11, 7, and 6, respectively (Supplementary Figure 2).

A.

Number of samples in Derivation datasets

			AF label 1		AF label 2		AF label 3	
			No. of patients	No. of samples	No. of patients	No. of samples	No. of patients	No. of samples
**Total**	AF label	----	***167***	----	***242***	----	***276***	----
	SR label	----	***1896***	----	***1896***	----	***1896***	----
**Training**	AF label	Patten B	***116***	116 **x11 x2** = **2552**	***170***	170 **x7 x2** = **2380**	***193***	193 **x6 x2** = **2316**
	SR label	Pattern A	***1326***	1326 ***x2*** = **2652**	***1326***	1326 ***x2*** = **2652**	***1326***	1326 ***x2*** = **2652**
**Validation**	AF label	Patten B	***17***	17 **x11 x2** = **374**	***24***	24 **x7 x2** = **336**	***28***	28 **x6 x2** = **336**
	SR label	Pattern A	***190***	190 ***x2*** = **380**	***190***	190 ***x2*** = **380**	***190***	190 ***x2*** = **380**
**Testing**	AF label	Patten A	***34***	34 **x2** = ***68***	***48***	48 **x2** = ***96***	***55***	55 **x2** = **110**
	SR label	Pattern A	***380***	380 ***x2*** = **760**	***380***	380 ***x2*** = **760**	***380***	380 ***x2*** = **760**

Supplementary Figure 2. Number of samples in each dataset: A. Derivation datasetes

B.

Number of samples in Extra testing datasets

			CNN algorithm 1		CNN algorithm 2		CNN algorithm 3	
			No. of patients	No. of samples	No. of patients	No. of samples	No. of patients	No. of samples
Extra testing dataset 1	AF label	Pattern A	***293***	293 ***x2*** = 586	***318***	318 ***x2*** = 636	***340***	340 ***x2*** = 680
	SR label	Patten A	***1516***	1516 ***x2*** = 3032	***1516***	1516 ***x2*** = 3032	***1516***	1516 ***x2*** = 3032
Extra testing dataset 2	AF label	Pattern A	***128***	----	***128***	----	***128***	----
	Length of time from the index AF-ECG (days)		No. of ECGs taken	No. of samples	No. of ECGs taken	No. of samples	No. of ECGs taken	No. of samples
	< -366		***85***	85 ***x2*** = 170	***85***	85 ***x2*** = 170	***85***	85 ***x2*** = 170
	-365 to -181		***62***	62 ***x2*** = 124	***62***	62 ***x2*** = 124	***62***	62 ***x2*** = 124
	-180 to -91		***49***	49 ***x2*** = 98	***49***	49 ***x2*** = 98	***49***	49 ***x2*** = 98
	-90 to -32		***43***	43 ***x2*** = 170	***43***	43 ***x2*** = 170	***43***	43 ***x2*** = 170
	-31 to -1		----	----	***57***	57 ***x2*** = 114	----	----
	0 to 31		***136***	136 ***x2*** = 272	----	----	----	----
	32 to 90		***55***	55 ***x2*** = 110	***55***	55 ***x2*** = 110	***55***	55 ***x2*** = 110
	91 to 180		***74***	74 ***x2*** = 148	***74***	74 ***x2*** = 148	***74***	74 ***x2*** = 148
	181 to 365		***87***	87 ***x2*** = 174	***87***	87 ***x2*** = 174	***87***	87 ***x2*** = 174
	≥366		***93***	93 ***x2*** = 186	***93***	93 ***x2*** = 186	***93***	93 ***x2*** = 186
			No. of patients	No. of samples	No. of patients	No. of samples	No. of patients	No. of samples
Extra testing dataset 3	SR label	Pattern A	***9,735***	----	***9,735***	----	***9,735***	----
	Length of time from the index AF-ECG (days)		No. of ECGs taken	No. of samples	No. of ECGs taken	No. of samples	No. of ECGs taken	No. of samples
	≤180		***8,013***	8,013 ***x2*** = 16,026	***8,013***	8,013 ***x2*** = 16,026	***8,013***	8,013 ***x2*** = 16,026
	181 to 365		***370***	370 ***x2*** = 7408	***370***	370 ***x2*** = 7408	***370***	370 ***x2*** = 7408
	366 to 730		***821***	821 ***x2*** = 1,642	***821***	821 ***x2*** = 1,642	***821***	821 ***x2*** = 1,642
	731 to 1094		***531***	531 ***x2*** = 1,062	***531***	531 ***x2*** = 1,062	***531***	531 ***x2*** = 1,062
	≥1095		----	----	----	----	----	----

Supplementary Figure 2. Number of samples in each dataset: B. Extra testing datasets

Reference

[1] Buda M, Maki A, Mazurowski MA. A systematic study of the class imbalance problem in convolutional neural networks. Neural Netw. 2018;106:249-59.

[2] Le Guennec A, Malinowski S, Tavenard R. Data Augmentation for Time Series Classification using Convolutional Neural Networks. ECML/PKDD Workshop on Advanced Analytics and Learning on Temporal Data. Riva Del Garda, Italy2016.

[3] Farias da Silva MA, De Carvalho RL, Almeida TdS. Evaluation of a Sliding Window mechanism as DataAugmentation over Emotion Detection on Speech. Academic Journal on Computing, Engineering and Applied Mathematics. 2021;2:11-8.

[4] Shenfield A, Howarth M. A Novel Deep Learning Model for the Detection and Identification of Rolling Element-Bearing Faults. Sensors (Basel). 2020;20:5112.
